# Cytocompatibility and Mechanical Properties of Short Phosphate Glass Fibre Reinforced Polylactic Acid (PLA) Composites: Effect of Coupling Agent Mediated Interface

**DOI:** 10.3390/jfb3040706

**Published:** 2012-10-16

**Authors:** Muhammad Sami Hasan, Ifty Ahmed, Andrew Parsons, Gavin Walker, Colin Scotchford

**Affiliations:** Division of Materials, Mechanics and Structures, Faculty of Engineering, University of Nottingham, University Park, Nottingham NG7 2RD, UK; Email: eaxmsh@nottingham.ac.uk (M.S.H); ifty.ahmed@nottingham.ac.uk (I.A.); andrew.parsons@nottingham.ac.uk (A.P.); gavin.walker@nottingham.ac.uk (G.W.)

**Keywords:** phosphate glass, fibre, PLA, composite, degradation, cytocompatibility, mechanical properties, coupling agent

## Abstract

In this study three chemical agents Amino-propyl-triethoxy-silane (APS), sorbitol ended PLA oligomer (SPLA) and Hexamethylene diisocyanate (HDI) were identified to be used as coupling agents to react with the phosphate glass fibre (PGF) reinforcement and the polylactic acid (PLA) polymer matrix of the composite. Composites were prepared with short chopped strand fibres (l = 20 mm, ϕ = 20 µm) in a random arrangement within PLA matrix. Improved, initial composite flexural strength (~20 MPa) was observed for APS treated fibres, which was suggested to be due to enhanced bonding between the fibres and polymer matrix. Both APS and HDI treated fibres were suggested to be covalently linked with the PLA matrix. The hydrophobicity induced by these coupling agents (HDI, APS) helped to resist hydrolysis of the interface and thus retained their mechanical properties for an extended period of time as compared to non-treated control. Approximately 70% of initial strength and 65% of initial modulus was retained by HDI treated fibre composites in contrast to the control, where only ~50% of strength and modulus was retained after 28 days of immersion in PBS at 37 °C. All coupling agent treated and control composites demonstrated good cytocompatibility which was comparable to the tissue culture polystyrene (TCP) control, supporting the use of these materials as coupling agent’s within medical implant devices.

## 1. Introduction

Over the last two decades research has focused on development of materials that may provide solutions for issues such as stress shielding, corrosion, implant-removal surgery and risk of secondary fractures associated with removal of metallic implants. Self­reinforced polymer composites have been fabricated with greater strength and improved Young’s modulus compared to the intrinsic properties of the polymer alone [1,2,3,4]. For example, self­reinforced poly-DL-lactide was found to have a moderate Young’s modulus (~12 GPa) and acceptable biocompatibility, which made them useful as fixation screws. However, their strength was found to be insufficient for fixation of load­bearing bone repair applications [2].

Recent work has explored resorbable fibre reinforced composites to be used as bone fracture fixation devices. The major biodegradable fibres, include plant-originated (natural) fibres (e.g., flax, hemp, jute, ramie, kenaf, abaca), alginate fibres, polymer fibres (e.g., polylactic acid and polyhydroxyalkanoate) and phosphate glass fibre (PGF). To improve the strength and stiffness (Young’s modulus) of degradable polymers ideally a degradable fibre with strength characteristics closer to E-glass fibre (strength ~2000 MPa; modulus ~80 GPa) [5] is required. PGFs are relatively easy to fabricate (depending on formulation) and exhibit high tensile strength (up to 450 MPa), high modulus (up to 80 GPa) and controllable degradation rates [6,7]. A number of studies have shown the potential of phosphate glass particulates or PGF reinforced resorbable polymer composites (see Table 1 for range of mechanical properties achieved). Although the initial strengths and modulus values of these composites were found to be sufficient for load­bearing applications, they experienced a rapid loss of mechanical properties when exposed to an *in vitro* aqueous environment [8,9,10]. Lin *et al*. tested PLLA reinforced with 55% (by volume) biodegradable calcium phosphate glass fibres. The initial mechanical properties of their samples with unidirectional fibres were well characterised: tensile, compressive, flexural and short beam shear strengths were reported as 200, 186, 161, and 19 MPa respectively, with tensile and flexural moduli recorded as 30 and 27 GPa. However, these samples only managed to retain 35% and 45% of their initial tensile strength and modulus after 23 days in phosphate buffered saline at 37 °C [11]. Rapid degradation or loss of mechanical properties from these composite implants could result in improper fixation of the fracture especially during the early stages of healing and thus compromise the bone repair process. 

**Table 1:** Summary of selected investigations on phosphate glass fibre reinforced composites and their mechanical properties. PCl: Poly-caprolactone, PLA: poly-lactic acid, POE: poly-orthoester, PGF: phosphate glass fibre, UD: unidirectional.

Matrix	Reinforcement	Flexural strength	Modulus	Reference
	V_f_ (%)	(MPa)	(GPa)	
PLA	Continuous UD and 10mm random Quinternary PGF (15‑20%)	106‑115	6.8‑9	[9]
POE	Short random ternary PGF (0–50%)	65–103	1.5‑9.4	[12]
PLA	10mm random quaternary PGF 14%	90	5	[13]
methacrylate-modified oligolactide	50 cm long quinternary PGF (NR)	115 ± 11.9	16 ± 2.4	[14]
PCL ( *in situ* polymerisation)	Continuous UD quaternary PGF 25%	105 ± 12	5.9 ± 6	[15]
PCL (compression moulding)	Continuous UD quaternary PGF 25%	55 ± 8	2.1 ± 0.3	[15]
PCL	Continuous UD quinternary PGF (10% wt)	72	2.74	[16]
methacrylate-modified oligolactide	30 cm quaternary PGF	110–190	15–20	[17]
PCL	10mm random binary PGF (6‑18%)	30	2.5	[18]
PLA	Continuous UD and short random PGF (40‑55%)	120–350	10 to 30	[19]

The rapid decrease in mechanical properties for these fully resorbable composites can be explained by two phenomena: (i) early hydration at the polymer/fibre interface could initiate de-bonding and create stress concentration sites, which would prevent the transfer of stress from the fibre to the polymer matrix; (ii) polymer swelling could generate hydrostatic forces that may crack the reinforcement phase [8,9,10]. Controlling the degradation of the fibre matrix interface and/or decreasing polymer swelling would be vital in order to manufacture resorbable composites that retained their mechanical properties for the desired period of time before resorption started.

Interfacial strength between the polymer matrix and the reinforcement phase can be improved by various means, including mechanical interlocking, plasma treatment or via chemical coupling. Coupling agents are more commonly employed to improve the interfacial properties [20,21,22,23], which can also in some cases improve the initial mechanical properties of the composite. It is hypothesised that introduction of covalent bonds and/or hydrophobicity at this interface could delay hydration of the fibre matrix interface and decrease polymer swelling, which would help reduce internal stress concentration sites within the system. Improving the interface would enable further control over the composite properties by providing tailored load transfer of the composite to the healing bone (potentially reducing stress shielding effects). It is also recognised that degradation products from the composites (including any potential coupling agents used) should not elicit a cytotoxic response.

Amino-propyl-triethoxy-silane (APS) has been used as a coupling agent within silicate glasses and a PGF reinforced composite [24,25]. Various researchers have reported the effectiveness of silanes on interfacial shear strength (IFSS) between silica based glasses and their respective polymer matrices [25,26,27]. For example, Park and Jin reported an increase in interlaminar shear strength (ILSS) between E-glass fibre and unsaturated polyester matrix when a combination of two silanes methacryloxypropyltrimethoxy silane (90 wt%, MPS) and aminopropyltriethoxy silane (10 wt%, APS) in methanol/distilled water (95/5 volume %) was used for surface treatment of silica glass fibres with different concentrations. It was reported that with 0.2 molar concentration, ILSS improved from ~16 MPa (control) to ~26 MPa [25]. However, the IFSS values ~8.9 MPa for the silane treated PGF obtained, were similar to that of the control (untreated glass fibre) [20,28]. Improvement in flexural strength and modulus values by ~25 MPa and ~1 GPa respectively, for PGF reinforced polyorthoester was reported with silane treatment [23]. Similarly a sorbitol ended PLA oligomer (SPLA) was reported to improve IFSS from 15 MPa to 23 MPa [28]. However, the effects of SPLA as a coupling agent on the mechanical properties of a composite have not been reported in the literature. Hexamethylene diisocyanate (HDI) has been reported as being acutely cytotoxic and an irritant to the skin and eyes. However, despite these reported toxicology issues there is also evidence for the effective use of HDI as a cross-linker in artiﬁcial extracellular matrix protein production genetically engineered from elastin and ﬁbronectin derived repeat units [29], a modifier in drug delivery systems a surface-modifier/coupler in biocomposites [30] and as a surface modifier of calcium phosphate ceramics [30,31,32,33] investigated for use as medical implants.

In this study the above three chemical agents (APS, SPLA and HDI) were identified and investigated as potential coupling agents to react with the PGF reinforcement and the PLA polymer matrix. Studies have shown that these agents improved IFSS properties and could potentially adjust the wettability of glass fibres by altering their surface chemistry from being extremely hydrophilic (with SPLA treatment) to very hydrophobic (with HDI treatment). This study investigates the effects of these coupling agents on the composite mechanical properties and *in vitro* cytocompatibility is also assessed using direct contact methods with human osteosarcoma cells. The composite manufacturing process and testing is discussed along with cytocompatibility studies conducted over a 7 day period.

## 2. Results and Discussion

It is well known that fibre reinforcement of polymer matrices can produce mechanically stronger materials. Despite all the variations of fibre volume fraction, degradable polymer matrix choice, fibre lay-out and fibre type, maximum initial flexural strength values of ~80 MPa to ~200 MPa and modulus values of ~5 GPa to 30 GPa have been reported by various authors [9,10,12,13,14,15,16,34]. However, these values were reported to decrease to ~40 MPa and 50 MPa and 1.2 GPa to 15 GPa strength and modulus respectively after approximately 4 weeks of degradation *in vitro*. This is too rapid a loss for practical application as orthopaedic implants especially as bone fracture repair plates. To investigate the hypothesis that coupling agents could increase the mechanical properties and also provide control over composite mechanical property loss *in vitro*, three coupling agents (with different wettability, from hydrophobic to hydrophilic) were employed and tested for their mechanical properties and cytocompatibility.

### 2.1. Flexural Mechanical Properties

**Figure 1:**
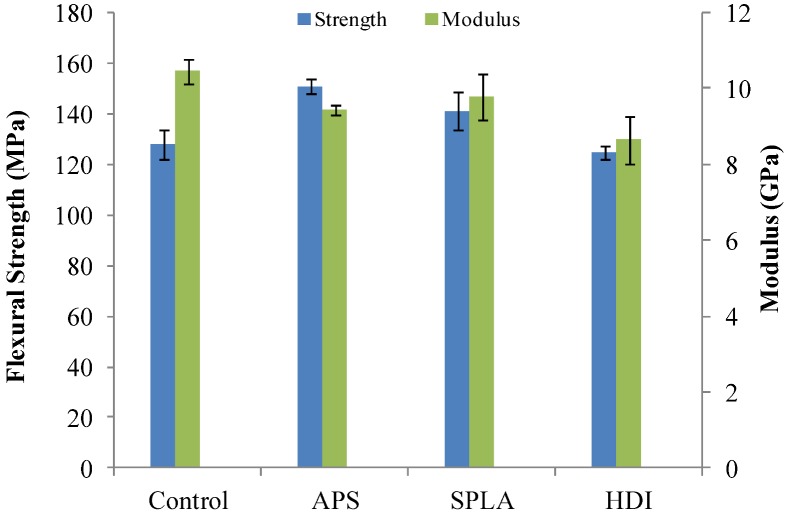
Mechanical properties obtained for the untreated and coupling agents treated non‑woven PGF reinforced PLA composites produced with approximate fibre volume fraction of 20%. Error bar represents standard error of mean where n = 3.

From the mechanical studies conducted (three­point bend), it was observed that the initial flexural strength of the composites increased by 20 MPa (7%) and ~15 MPa (5.5%) with APS and SPLA treatment respectively in comparison with untreated control samples (see Figure 1). However, a decrease of ~5 MPa (2.5%) was seen for HDI treated samples. The flexural modulus was found to slightly decrease from 10 GPa (for the untreated control composite) to ~8–9 GPa for all coupling agent treated PGF reinforced composites (see Table 2). However, statistical analysis revealed no significant difference (*p* > 0.05) between the initial mechanical properties of the control and coupling agent (APS, SPLA and HDI) treated samples investigated.

**Table 2:** Fibre volume fraction for composites as measured by matrix burn out test. Values after ± represents standard deviation with n = 3.

Sample code	Fibre volume fraction (%)
Control	20 ± 4
APS	23 ± 6
SPLA	21 ± 5
HDI	18 ± 3

A study investigating APS treatment of PGF reinforced polycaprolactone showed improvement of flexural properties from ~2 GPa (modulus) and ~50 MPa (strength) to ~3.5 GPa (modulus) and ~80 MPa (strength) [35]. Another study on PCL based composites reinforced with PGFs treated with (poly 2-hydroxyethyl methacrylate) poly-HEMA revealed an improvement of 10 MPa in flexural strength. The authors attributed the improvements seen to better shear bond strength, chemical coupling and/or concealing of micro­cracks between the phases. 

The ethoxy functionality in APS is believed to condense with the hydroxyl functionality on the surface of the glass fibre whilst the amine functionality can react with the polymer matrix [24,36]. It is suggested that HDI was grafted to the surface of the glass fibre and polymer via covalent bonds as follows: glass-O-CO-NH-(CH_2_)_6_-N=C=O. The urethane linkage between the glass surface and HDI converts into a primary amine group after treatment with water which can bind with the polymer through a covalent link [30]. The PLA-oligomer end functional groups (-OH in the case of sorbitol) has the potential to react with the glass through the hydroxyl groups present on the glass surface [20,21,28]. These reactions are believed to improve IFSS and as a result infer better load transfer from the polymer matrix to glass fibre which results in improved mechanical properties of the resultant composite. 

The lack of improvement in mechanical properties observed here for the HDI treated composites could potentially be due to the relatively lower fibre volume fraction and loss of fibre strength during the HDI treatment process for PGFs, which involved soaking of the fibres in water for 15 minutes. 

**Figure 2:**
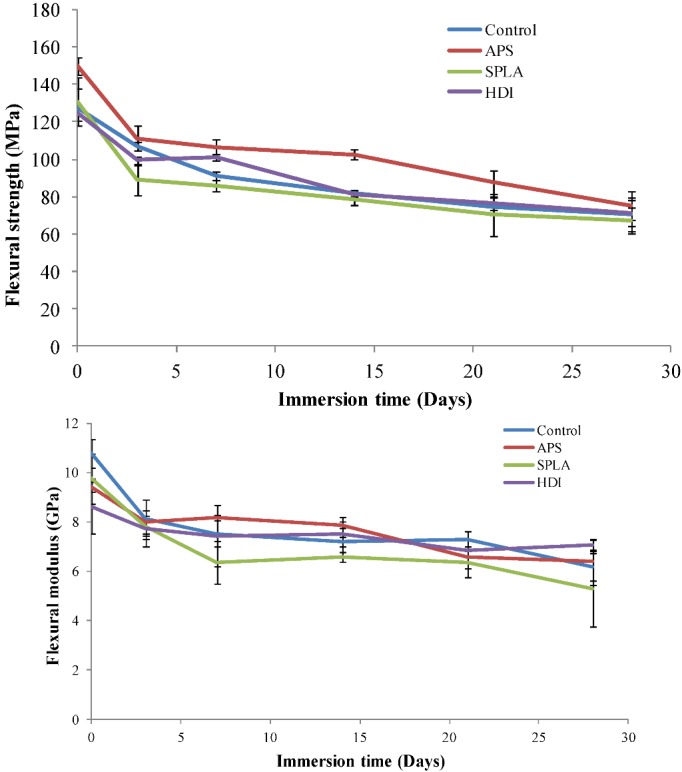
Retention of mechanical properties with degradation obtained for the untreated and coupling agent treated non-woven PGF reinforced PLA composites; (**a**) flexural strength; (**b**) flexural modulus. Error bar represents standard deviation where n = 3.

Although, it appeared that an apparent improved strength and modulus retention for APS and HDI treated samples could be observed in Figure 2 (a,b), the statistical analysis (for samples degraded in phosphate buffered saline at 37 °C for up to 28 days) revealed no significant difference (*p* > 0.05) between all the samples at any time point. However, a rapid loss of properties was observed from the control and SPLA treated composite samples in comparison. The loss observed for SPLA was suggested to be due to the hydrophilic nature of the oligomer [37], which absorbed significantly large amounts of water and thus lost interfacial integrity due to hydrolysis of the hydrogen bonds between the PLA oligomer and glass fibre surface. 

After 28 days immersion in PBS an approximate 50% mechanical strength decrease was seen for the control, APS and SPLA treated samples. However, the HDI treated samples retained significantly greater strength (approximately 67%) in comparison to their initial strength over the 28 day degradation period. Similarly a decrease of ~50% for modulus was seen for control and SPLA treated samples (in comparison to their day 1 values) whilst APS and HDI treated composite samples retained ~65% and ~70% of their initial modulus after 28 days of immersion respectively. As stated earlier the final values were found to be not statistically significant; however, after 28 days of immersion the retention % for APS (modulus) and HDI (strength and modulus) was observed to be higher. The improved retention of modulus seen for HDI and APS treated composites was attributed to strong covalent bonds between PGFs and the PLA matrix enhanced by their hydrophobicity at the interface. It was also observed that SPLA treated samples swelled after immersion in PBS due to the hydrophilic nature of SPLA [37]. Rapid loss of mechanical properties for these SPLA treated samples was suggested to be due to greater water penetration which accelerated the hydrolysis of hydrogen bonds between the oligomer and the PGFs. 

Mechanical properties of a polymer are (up to a certain molar mass) directly proportional to the chain length. Therefore mechanical property retention of polyester (such as PLA, PGA or PCL) based composites can also depend on the degradation of the polymer via hydrolysis of the ester group in the polymer backbone. Hydrolysis of polyesters is catalysed by protons (*i.e.*, in an acidic environment). PLA has been known to hydrolyse more readily than other aliphatic polyesters. The acid dissociation constant (pKa) of oligomeric PLA is 3.1. Therefore, the dissociation of the acid end-group is expected to result in an acidic environment and contribute significantly toward acid-catalysed hydrolysis. As the reaction proceeds, the carboxylic acid concentration and the rate of hydrolysis increase, and the reaction is said to be autocatalytic [38], which can result in loss of mechanical properties of the polymer. This also highlights that the approach to reduce hydration (by improving the fibre/PLA interface) is therefore likely to improve mechanical performance not only by prevention of swelling and cracking, but by also delaying polymer hydrolysis. The effect of autocatalysis of the polyester matrix was reported to significantly affect the mechanical properties of PGF reinforced composite [17]. 

### 2.2. SEM Analysis of PGF/PLA Interface

**Figure 3:**
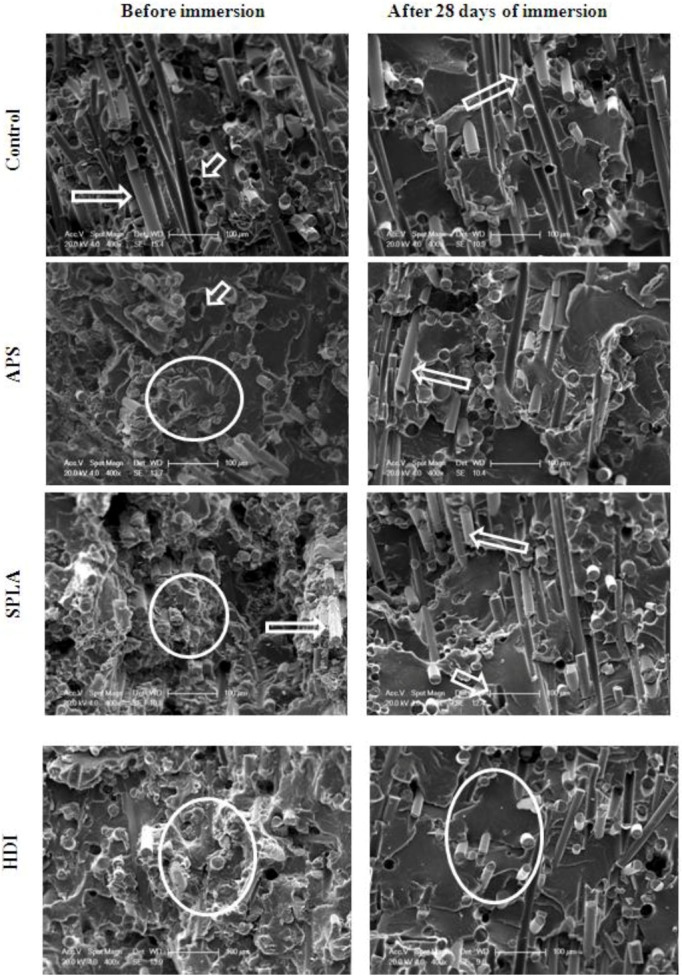
SEM micrographs from top to bottom: untreated (control) composite APS treated, SPLA treated, HDI treated random PGF reinforced PLA composite, before (left) and after (right) 28 days of immersion in PBS at 37 °C. Micrometer scale-bar = 100µm. Arrows indicate holes and fibre pull-out due to interface failure and circles highlight intact interfacial integrity and short fibre lengths. Better interface *i.e.*, shorter fibre length was observed in case of HDI treated composites compared to others after 28 days of immersion.

Cross sections of the composites were exposed by freeze/fracture and examined via SEM, Figure 3(a–d) shows representative images of all the samples as made and after 28 days degradation in PBS at 37 °C. It can be observed from Figure 3a to Figure 3d (Control, APS, SPLA and HDI treated composite respectively) that for composites treated with coupling agents (*i.e.*, for non-degraded composites) the fibres show good wet-out with the polymer matrix. For the untreated composite samples clean long fibres and holes were seen which was suggestive of a weak interface between PGFs and PLA. This correlated well with the difference observed for initial flexural mechanical properties and their retention. No obvious differences between the SPLA, APS treated and control composites were noticed from SEM analysis for samples after 28 days of immersion. However, it was noticed that the HDI treated samples appeared to maintain their interface to some degree, which in turn further supported the data above which apparently showed improved retention of mechanical properties in comparison with the other samples tested. 

### 2.3. Cytocompatibility Analysis

For a material to be used as an implant within the body it needs to satisfy biocompatibility assessment. Culturing cells directly on the surface of composites may also indicate synergistic interaction of cells with PLA, PGF and coupling agents. The chemical agents used in this project were selected on the basis of their potential or reported cytocompatibility. For example, Sánchez­Vaquero *et al*. [39] and Jung *et al*. reported favourable cell interaction with silane containing biomaterials. However, conflicting reports of cytotoxicity or cytocompatibility have been reported for HDI [30,31,32,40] and no data could be found in the literature reporting on the cytocompatibility for SPLA oligomer. To ascertain the cytocompatibility of the materials used in this project a bone-like cell line (MG63 osteosarcoma) was cultured onto the surface of the composites. Cell functions such as metabolism, viability, proliferation and early differentiation markers were evaluated. Cell morphology was investigated using SEM.

**Figure 4:**
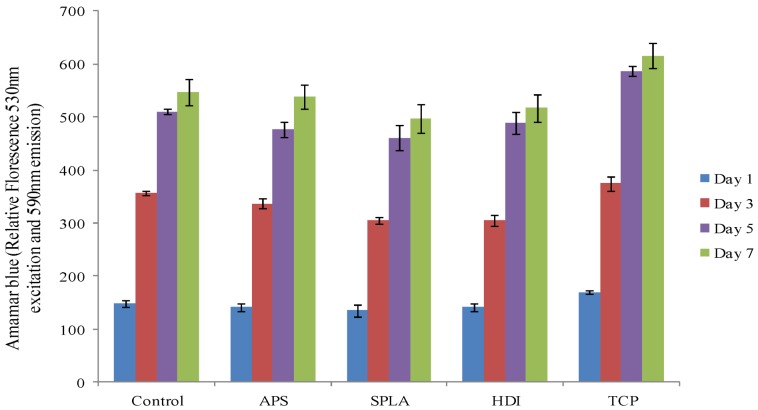
Metabolic activity of MG63 osteosarcoma, as measured by the alamar blue assay, cultured on PGF/PLA composites, x-axis represents surface treatments. Error bar represents standard error of mean, n = 6.

Metabolic activity was seen to increase up to day 5 of the culture period and then levelled off at the seven day interval. However, there was no significant difference in metabolic activity found amongst the different composite samples (control, APS, SPLA, HDI) investigated at any time point (*p* > 0.05). Notably at the 5th and 7th day of culture the tissue culture polystyrene (TCP) control demonstrated an elevated metabolic activity compared to the composite samples that were investigated (Figure 4). 

**Figure 5:**
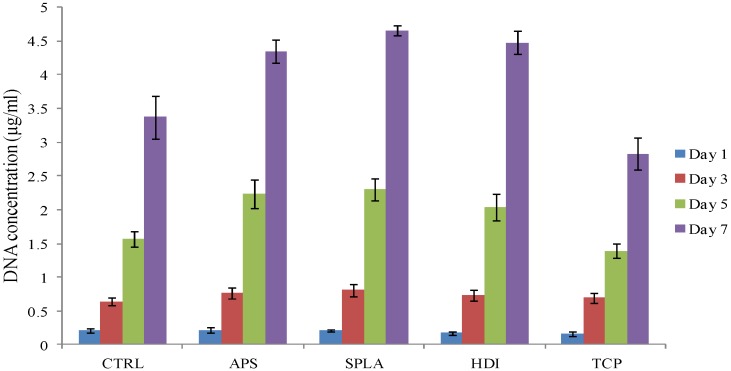
Cell proliferation of MG63 osteosarcoma, as measured by the DNA (Hoechst 33258) assay, cultured on PGF/PLA composite, x-axis represents surface treatments. Error bar represents standard error of mean, n = 6.

The DNA content of the cells cultured on composite samples was used as an indicator of cell population. DNA (Hoechst 33258) assay was used to quantify the changing concentration of DNA with time for MG63 osteosarcoma cells, (see Figure 5).

DNA concentration after one day was approximately 0.2 μg mL^−1^ for all surfaces. The levels of DNA gradually and continuously increased over the period of culture. There was no statistically significant difference (*p* > 0.05) found between the final DNA concentrations on any composite samples. However, DNA concentration observed for TCP was significantly lower than for the composite samples at day 5 and 7. Cells cultured on TCP were found to be relatively large in size less dense as seen by SEM imaging (Figure 3) which could have resulted in lower cell number and hence DNA concentration on TCP. 

**Figure 6:**
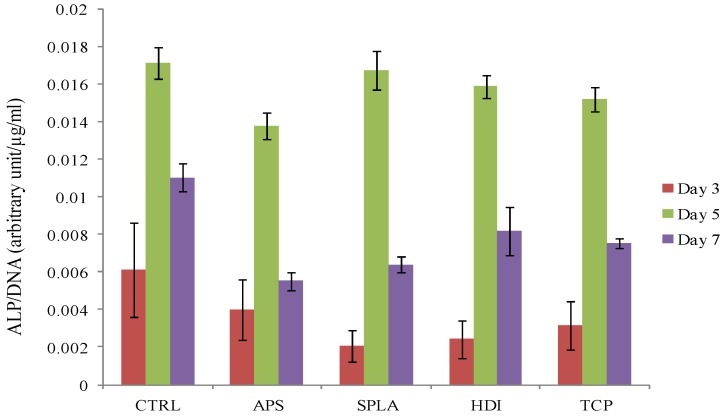
Alkaline phosphatase (ALP) activity of MG63 osteosarcoma cells, as measured by ALP assay, cultured on PGF/PLA composite, x-axis represents surface treatments. Data was normalised with corresponding DNA concentration for each individual sample.

The effect of coupling agent treatments on cell phenotype was analysed by measuring the alkaline phosphatase activity of osteosarcoma cells cultured on composite discs with different coupling agent treatments. Data was normalised with the corresponding DNA concentration at each time point (Figure 6).

For all surfaces, including the TCP positive control, the alkaline phosphatase activity was not detectable after 1 day of cell culturing. However, after 3 days of culture detectable amounts of ALP were observed on all the surfaces, which increased at day 5. No statistically significant difference (*p* > 0.05) was observed between the composite samples and control. Although the concentration of ALP increased with culture time, the ALP/DNA ratio decreased from day 5 to day 7 following the standard up and down regulation of ALP with culture time [41]. 

The response of osteoblast like cells cultured directly onto a composite surface was investigated, bearing in mind that the surface properties, combination of degradation products and their concentration from the composite would be different to those for the polymer, glass and coupling agents alone.

A small number of studies have reported on the cytocompatibility of PLA based composites with various reinforcements like HA, Bioglass or phosphate glass in comparison to tissue culture polystyrene [12,13,14,42,43]. For example, human osteosarcoma cells (line MG63) cultured for 7 days on annealed and non‑annealed phosphate glass fibre reinforced PLA composites (~14% by volume) were imaged using live/dead (calcein AM / propidium iodide stain) staining. It was reported that both PLA (alone) and the annealed fibre reinforced composites maintained higher cell viability as compared to the non­annealed fibre composites. This was attributed to the slower degradation rate of the annealed fibre reinforced composites [13]. Brauer *et al* produced an *in-situ* polymerised polymer matrix supplemented with methacrylic acid 2-hydroxyethylester (10 wt%) as a co-monomer, reinforced with slow degrading phosphate invert glass fibres of the glass system P_2_O_5_­CaO­MgO­Na_2_O­TiO_2._ MC3T3­E1 murine pre-osteoblast cell were seeded on cross-sections of the prepared samples and cultured for up to 8 days. Short­term biocompatibility was tested in an FDA/EtBr viability assay and good cell compatibility for the composite materials was reported on the basis of live­cell density attached to the surfaces [14].

Potential issues with short chain PLA and HDI have been reported. Ignatius and Claes reported that high concentrations of degradation products from PLA had a toxic influence on the cell culture systems when studied with MTT and BrdU assays [44]. In another study increased inflammation was reported when the molecular weight of PLA/PGA copolymers decreased to 10,000–20,000 [45]. It was also reported that toxic hexamethylene diamine (HDA) could be released [30] from HDI grafted hydroxyapatite. However, in this study all coupling agent treated and control composites demonstrated cytocompatibility comparable to TCP control supporting their use within implantable resorbing devices.

**Figure 7:**
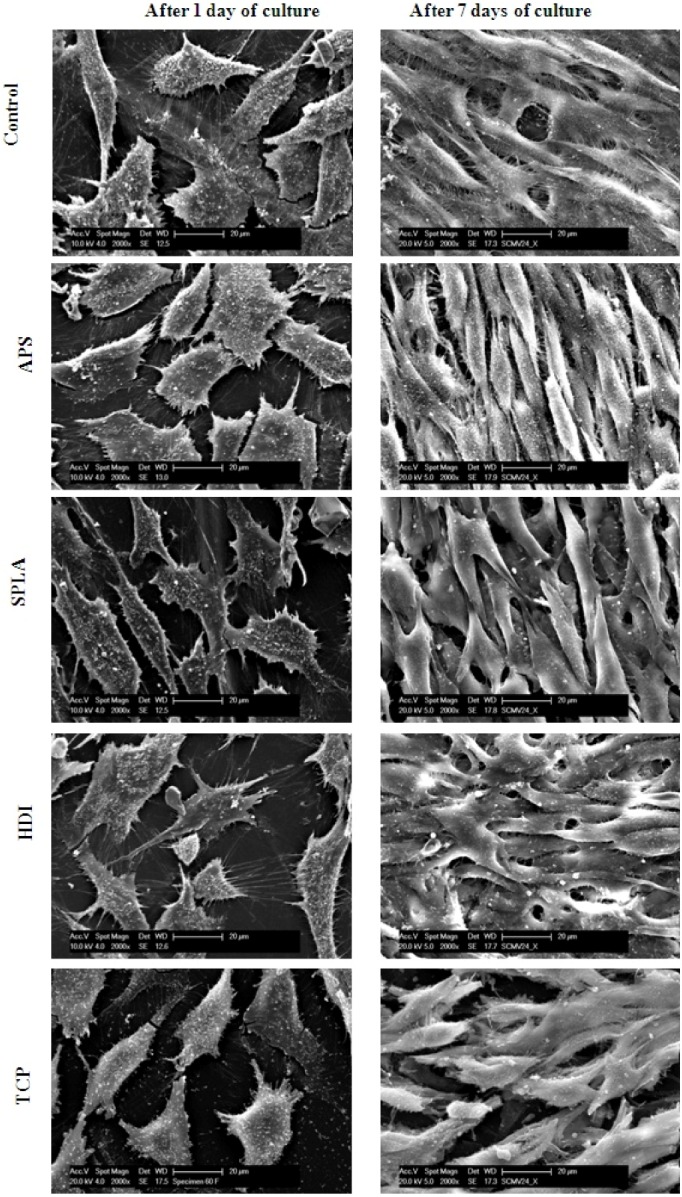
SEM micrographs of MG63 cells cultured on, untreated (control) random, APS treated, SPLA treated, HDI treated random PGF reinforced PLA composite and TCP for 1 (left) and 7 (right) days. Micrometer bar = 20 µm

SEM images of MG63 osteosarcoma cells (Figure 7a–e) revealed that the cell morphology was almost identical on all surfaces (composite and TCP) at day 1 and day 7 of culture. Cells appeared to show many filapodia and lamellipodia attached to the surface at day 1 and a multi-layered cell matrix could be observed at day 7. However, cells on the SPLA treated composite appeared smoother than other samples by the 7th day of culture. Cell morphology and cell number from the SEM images correlated well with the observations of viability, proliferation and differentiation markers above.

A comparison of results obtained for flexural mechanical properties and their retention in this study to similar reports [9,10,12,13,14,15,16,46] indicates that a composite with enhanced ability to retain its mechanical integrity has been produced by virtue of chemical coupling provided by HDI and APS. After 4 weeks of degradation *in vitro,* flexural mechanical properties in the rage of ~40 MPa to 50 MPa (strength) and 1.2 GPa to 15 GPa (modulus) have been reported [9,10,12,13,14,15,16]. The composites produced in this study were found to have initial strengths and moduli in the range of ~125 MPa to ~150 MPa and ~10 GPa respectively. After four weeks of degradation *in vitro,* the best composite (HDI treated composite) was found to retain ~70% of initial strength and modulus. The initial improvement was attributed to improved interfacial shear strength. The retention of mechanical integrity was also attributed to better interfacial shear strength as well as hydrophobicity of the interface. Initial *in vitro* biocompatibility assessment suggests that the coupling agent treatment does not have a negative effect on the biological responses in terms of osteoblast like cell functions or morphology. With all the improvements achieved, mechanical properties of the composite are still closer to the lower limits of the targeted mechanical properties of cortical bone (bending strength 100–200 MPa, E = 10–30 GPa). Therefore, further improvement in terms of fibre tensile properties, polymer choice (more hydrophobic), fibre volume fraction, coupling agent treatment with optimised concentrations and composite design are required. 

## 3. Experimental Section

### 3.1. Glass Synthesis

Phosphate glass (45P_2_O_5_ 16CaO 24MgO 11Na_2_O 4Fe_2_O_3_ in molar %) was prepared using the following precursors: NaH_2_PO_4_, CaHPO_4_, MgHPO_4_.3H_2_O, P_2_O_5_ and FePO_4_.2H_2_O (Sigma Aldrich, Dorset, UK). The precursors were weighed and mixed into a Pt/5% Au crucible type BC18 (Birmingham Metal Company, Birmingham, UK), which was then dried in a furnace at 350 °C for 30 minutes, before being transferred to another furnace to be melted at 1100 °C for 90 minutes. 

### 3.2. Fibre Production

PGF with an average diameter of 20µm were produced by melt­draw spinning using a dedicated in­house facility. Fibres were collected on a 1m circumference drum covered with a PTFE sheet. Fibre drawing temperature and drum speed were adjusted to 1200–1300 °C and ~2000 rpm respectively. The fibres were removed from the drum and annealed using the following steps: temperature was ramped to 250 °C at a rate of 20 °C/minute; then ramped again from 250 °C to 475 °C (Tg-5 °C) at 1 °C/minute; hold for 90 minutes; then ramped down to 350 °C at a rate of 0.25 °C/min; left to cool down to 25 °C at rate of 1 °C/minute and finally hold the fibres at room temperature (in desiccators) for 24 hours before use.

### 3.3. Fibre Coupling Agent Treatment

All the chemicals except the PLA oligomers were bought from Sigma Aldrich (UK) and used without further modification. The PLA oligomers were synthesised following the protocol reported elsewhere [13]. The molecular weight and the polydispersity of the SPLA was observed to be ~2100 (Mn) and 2.1 (PDI). Twenty millimetre chopped strand glass fibres were treated with coupling agents by dip coating in 0.2 M APS solution in 90% ethanol 10% water or 0.25 M SPLA oligomer solution in chloroform or 0.2 M HDI solution in dimethylformamide. Treated fibres were washed three times with the corresponding solvent after treatment to wash away any unbound coupling agent. 

### 3.4. Fibre Mat Production (Air Lay)

Non-woven chopped strand random fibre mats were manufactured using 20 mm chopped strand PBG fibres. Untreated fibres (as control) or coupling agent treated glass fibres were dispersed within an air­lay rig using high pressure air. The air­lay rig setup is connected to a vacuum pump and a desired amount of loose fibres is gradually fed into the rig. Expanding laminar flow causes the fibres to spread out and deposit onto metal meshes (with 200 µm size pores), forming non­woven random fibre mats. The rig was opened and the fibre mats were removed along with the metal meshes. These unbound fibre mats were then sprayed with PLA/chloroform solution (2.5%) in order to bind the fibres together.

### 3.5. PLA Sheet Preparation

Approximately 0.2 mm thick PLA films were prepared via compression moulding 5g of PLA pellets (3251­D NatureWorks, Plc. average Mw 90,000–120,000 Dalton, PDI 1.6, T_m_ 170 °C, T_g_ 61 °C). The pellets were placed between two metallic plates and heated to 210 °C in a J.R. Dare heated press for 10 minutes, before being compressed at 3 bar pressure for 30 seconds. The plates were then cooled immediately by transferring to a cold (room temperature) press (Daniels UK) at the same pressure. The PLA pellets were dried in a vacuum oven at 50 °C for 48 hours prior to use.

### 3.6. Composite Production

Composites were prepared using a laminate film stacking process. 7 PLA films and 6 PGF random mats were stacked alternately within a 170 mm diameter, 1.6mm thick mould cavity which was then placed between two metallic plates covered with a PTFE sheet. This stack was then heated in the press for 15 minutes at 210 °C and pressed for 15 minutes at 38 bar. The plates were transferred to a second press and allowed to cool to room temperature at 38 bar for 15 minutes. The resulting laminated composites were cut using a band saw into 32 mm length, 15mm width samples for mechanical testing. The fibre volume fraction of the composites was obtained using the matrix burn off method, according to the standard test method ASTM D2584­94. The target fibre volume fraction of the composites was approximately 20%. 

### 3.7. Flexural Mechanical Properties Analysis

The strength and modulus were evaluated by flexural (three­point bend) testing using a Hounsfield Series S testing machine. Specimens cut from composite plates were placed individually into 30 ml glass vials. The vials were filled with PBS (pH ~7.4 ± 0.2) and maintained at a temperature of 37 °C. Initial (as made and dry) and degraded (degraded up to 28 days and blot dried) samples were tested. The degradation media was refreshed at regular time intervals to ensure a constant pH (~7.4). These measurements were done according to the standard BS EN ISO 14125:1998. A crosshead speed of 1mm/min and a 1 kN load cell was used. The measurements were carried out in triplicate (n = 3). 

### 3.8. SEM Analysis of Composite Cross-Section

Composite samples were degraded in phosphate buffered saline at 37 °C for 28 days. Cross sections of degraded and non­degraded composites were exposed by freeze-fracture in liquid nitrogen and analysed under XL 30 scanning electron microscope (Philips, UK) at an accelerating voltage of 10 kV. This technique does not represent actual (ductile) fracture behaviour of polymer-composite. However, a brittle fracture of polymer matrix is a common method employed to examine the interface between glass fibre and polymer matrices. 

### 3.9. Cytocompatibility Analysis

MG63 cells (human osteosarcoma) obtained from the European collection of cell cultures (ECACC) were cultured in complete Dulbecco’s modified eagle media (CDMEM) consisting of Dulbecco’s modified eagle media supplemented with 10% foetal calf serum (FCS), 2% HEPES buffer, 2% penicillin/streptomycin, 1% glutamine, 1% non­essential amino acids (Gibco Invitrogen, UK) and 0.85mM of ascorbic acid (Sigma Aldrich, UK). Cells were cultured in 75cm^3^ flasks (Falcon, Becton, Dickinson and Company; UK) at 37 °C in a humidified atmosphere with 5% CO_2_. Once confluent the cells were dissociated from the flask using trypsin/EDTA solution (a sterile, phosphate buffered saline solution (1×) containing 0.025% trypsin and 0.01% EDTA) and centrifuged at 1200 rpm for 4 minutes to produce a pellet, which was resuspended in fresh media. Cell concentrations were determined using a haemocytometer; viable cells were identified using trypan blue exclusion.

Composite samples (9 mm diameter, 1.6 mm thick discs) were sterilised in 70% ethanol for 30 minutes and washed three times with sterilised PBS prior to cell culture. Tissue culture polystyrene (TCP) was used as a positive control for cell growth. Cells were seeded onto the disc sample surfaces at a concentration of 40,000 cells/cm^2^ and were incubated at 37 °C in a humidified atmosphere with 5% CO_2_ for 2, 48, 96 and 168 hours. 

#### 3.9.1. Cell Viability/Metabolic Activity

At the designated time points, culture medium was removed from the wells and the samples were washed three times with warm PBS. Alamar Blue solution (1:9 Alamar Blue: warm hanks balanced salt solution (HBSS)) (1 mL) was added to each well and incubated for 90 minutes. From each well 100 µL aliquots were transferred to a 96 well­plate in triplicate and fluorescence was measured at 530 nm excitation and 590 nm emission using an FLx800 microplate reader (BioTek Instruments Inc., Bedford, UK).

#### 3.9.2. DNA Quantification

Samples were washed and cells lysed using the freeze/thaw technique. One hundred micro litre aliquots of cell lysate were transferred to a 96­well plate. DNA standards were prepared using calf thymus DNA (Sigma, UK) and TNE buffer (10mM Tris, 2M NaCl, and 1mM EDTA in deionised water, adjusted to pH 7.4) as diluents. One hundred micro litres of Hoechst stain 33258 was added to each well (1mg of bis-benzimide 33258 in deionised water, further diluted to 1:50 in TNE buffer) and the plate agitated. Fluorescence was measured at 360 nm excitation and 460 nm emission using an FL×800 microplate fluorimeter (BioTek Instruments Inc). DNA concentrations were derived from a standard curve generated by the software (KCjunior).

#### 3.9.3. Alkaline Phosphatase Activity

Alkaline phosphatase activity was measured using the Granutest 25 alkaline phosphatase assay (Randox, London, UK). At the designated time points, cell culture media was removed and the samples were washed three times with warm PBS prior to the addition of 1mL deionised water to each well. Cells were lysed using a freeze/thaw technique three times. A 50 µL aliquot of cell lysate was added to a 96-well plate along with 50µL of the alkaline phosphatase substrate (p­nitrophenyl phosphate in diethanolamine HCI buffer, pH 9.8), shaken gently and the absorbance measured at wavelengths of 405 and 620nm using an EL×800 microplate colorimeter (BioTek Instruments Inc., Bedford, UK).

#### 3.9.4. Cell Morphology Analysis

Samples were washed with warm PBS at 37 °C and fixed in 3% glutaraldehyde in 0.1 M Na-cacodylate buffer for 30 minutes, after 30 minutes fixative was replaced by a 7% sucrose in 0.1 M Na-cacodylate solution. Fixed samples were then washed twice in 0.1M cacodylate buffer, and post fixed in 1% osmium tetroxide in PBS for 45 minutes in a fume cupboard. Samples were dehydrated through a graded ethanol series (20, 30, 40, 50, 60, 70, 80, 90, 96 and 100% in water) for approximately 5 minutes each. Samples were then dried via hexamethyldisilazine before being sputter coated in gold or platinum and viewed with a Philips XL30 scanning electron microscope operated at 10 kV.

### 3.10. Statistical Analyses

Average values and standard deviation were computed for two iterations of the experiments. Statistical analysis was performed using the Prism software package [47]. Two­way analysis of variance (ANOVA) was calculated with the bonferroni post­test to compare the significance of change in one factor with time. The error bars presented represent standard error of mean with n = 3 for mechanical tests and n = 6 for the cytocompatibility study.

## 4. Conclusions

Selected coupling agents (APS, SPLA oligomer) improved initial flexural properties of random short fibre (l = 20 mm, ϕ = 20 µm) reinforced PLA composites by ~20 MPa. This improvement in initial flexural properties was associated with improved shear bond strength at the interface due to covalent bridging between glass fibres and polymer matrix provided by chemical coupling. Covalently linked hydrophobic coupling agents (HDI, APS) also helped retain mechanical properties for an extended period by delaying hydrolysis of the interface. A maximum of ~70% of initial strength and 65% of initial modulus was retained by the HDI treated composite in contrast to control where ~50% of strength and modulus decrease was observed. All coupling agent treated and control composites demonstrated cytocompatibility comparable to TCP control supporting their use within implantable resorbable devices.

## References

[1] Törmälä P. (1992). Biodegradable self-reinforced composite materials; Manufacturing structure and mechanical properties. Clin. Mater..

[2] Törmälä P., Pohjonen T., Rokkanen P. (1998). Bioabsorbable polymers: Materials technology and surgical applications. J. Eng. Med..

[3] Törmälä P., Vasenius J., Vainionpää S., Laiho J., Pohjonen T., Rokkanen P. (1991). Ultra-high-strength absorbable self-reinforced polyglycolide (SR-PGA) composite rods for internal fixation of bone fractures: *In vitro* and *in vivo* study. J. Biomed. Mater. Res..

[4] Enislidis G., Lagogiannis G., Wittwer G., Glaser C., Ewers R. (2005). Fixation of zygomatic fractures with a biodegradable copolymer osteosynthesis system: Short- and long-term results. Int. J. Oral Maxillofac. Surg..

[5] Wambua P., Ivens J., Verpoest I. (2003). Natural fibres: Can they replace glass in fibre reinforced plastics?. Compos. Sc. Technol..

[6] Ahmed I., Collins C.A., Lewis M.P., Olsen I., Knowles J.C. (2004). Processing, characterisation and biocompatibility of iron-phosphate glass fibres for tissue engineering. Biomaterials.

[7] Felfel R., Ahmed I., Parsons A., Harper L., Rudd C. (2012). Initial mechanical properties of phosphate-glass fibre-reinforced rods for use as resorbable intramedullary nails. J. Mater. Sci..

[8] Ramsay S., Pilliar R., Yang L., Santerre J. (2005). Calcium polyphosphate/polyvinyl acid-carbonate copolymer based composites for use in biodegradable load-bearing composites for orthopaedic implant fabrication. Key Eng. Mater..

[9] Felfel R.M., Ahmed I., Parsons A.J., Haque P., Walker G.S., Rudd C.D. (2012). Investigation of crystallinity, molecular weight change, and mechanical properties of PLA/PBG bioresorbable composites as bone fracture fixation plate. J. Biomater. Appl..

[10] Felfel R.M., Ahmed I., Parsons A.J., Walker G.S., Rudd C.D. (2011). *In vitro* degradation, flexural, compressive and shear properties of fully bioresorbable composite rods. J. Mech. Behav. Biomed. Mater..

[11] Lin T.C. (1986). Totally absorbable fiber reinforced composite for internal fracture fixation devices. Trans. Soc. Biomater..

[12] Andriano K.P., Daniels A.U., Heller J. (1992). Biocompatibility and mechanical properties of a totally absorbable composite material for orthopaedic fixation devices. J. Appl. Biomater..

[13] Ahmed I., Cronin P.S., Neel E.A.A., Parsons A.J., Knowles J.C., Rudd C.D. (2009). Retention of mechanical properties and cytocompatibility of a phosphate-based glass fiber/polylactic acid composite. J. Biomed. Mater. Res. B Appl. Biomater..

[14] Brauer D., Rüssel C., Vogt S., Weisser J., Schnabelrauch M. (2008). Degradable phosphate glass fiber reinforced polymer matrices: Mechanical properties and cell response. J. Mater. Sci. Mater. Med..

[15] Khan R.A., Parsons A.J., Jones I.A., Walker G.S., Rudd C.D. (2009). Surface treatment of phosphate glass fibers using 2-hydroxyethyl methacrylate: Fabrication of poly(caprolactone)-based composites. J. Appl. Polym. Sci..

[16] Khan R.A., Parsons A.J., Jones I.A., Walker G.S., Rudd C.D. (2010). Preparation and characterization of phosphate glass fibers and fabrication of poly(caprolactone) matrix resorbable composites. J. Reinf. Plast. Compos..

[17] Kobayashi H.S., Brauer D.S., Rüssel C. (2010). Mechanical properties of a degradable phosphate glass fibre reinforced polymer composite for internal fracture fixation. Mater. Sci. Eng. C.

[18] Ahmed I., Parsons A.J., Palmer G., Knowles J.C., Walker G.S., Rudd C.D. (2008). Weight loss, ion release and initial mechanical properties of a binary calcium phosphate glass fibre/PCL composite. Acta Biomater..

[19] Parsons A.J., Ahmed I., Haque P., Fitzpatrick B., Niazi M.I.K., Walker G.S., Rudd C.D. (2009). Phosphate glass fibre composites for bone repair. J. Bionic Eng..

[20] Haque P., Barker I.A., Parsons A., Thurecht K.J., Ahmed I., Walker G.S., Rudd C.D., Irvine D.J. (2010). Influence of compatibilizing agent molecular structure on the mechanical properties of phosphate glass fiber-reinforced PLA composites. J. Polym. Sci. A Polym. Chem..

[21] Haque P., Parsons A.J., Barker I.A., Ahmed I., Irvine D.J., Walker G.S., Rudd C.D. (2010). Interfacial properties of phosphate glass fibres/PLA composites: Effect of the end functionalities of oligomeric PLA coupling agents. Compos. Sci. Technol..

[22] Dupraz A.M.P., Wijn J.R.d., Meer S., Groot K. (1996). Characterization of silane-treated hydroxyapatite powders for use as filler in biodegradable composites. J. Biomed. Mater. Res..

[23] Andriano K.P., Daniels A.U., Heller J. (1992). Effectiveness of silane treatment on absorbable microfibers. J. Appl. Biomater..

[24] Dibenedetto A.T., Lex P.J. (1989). Evaluation of surface treatments for glass fibers in composite materials. Polym. Eng. Sci..

[25] Park S.-J., Jin J.-S. (2003). Effect of silane coupling agent on mechanical interfacial properties of glass fiber-reinforced unsaturated polyester composites. J. Polym. Sci. B Polym. Phys..

[26] DiBenedetto A.T. (2001). Tailoring of interfaces in glass fiber reinforced polymer composites: A review. Mater. Sci. Eng. A.

[27] Yazdani H., Morshedian J., Khonakdar H.A. (2006). Effects of silane coupling agent and maleic anhydride-grafted polypropylene on the morphology and viscoelastic properties of polypropylene-mica composites. Polym. Compos..

[28] Haque P. (2011). Oligomeric PLA Coupling Agents For Phosphate Glass Fibres/PLA Composites.

[29] Nowatzki P.J., Tirrell D.A. (2004). Physical properties of artificial extracellular matrix protein films prepared by isocyanate crosslinking. Biomaterials.

[30] Dong G.-C., Sun J.-S., Yao C.-H., Jiang G.J., Huang C.-W., Lin F.-H. (2001). A study on grafting and characterization of HMDI-modified calcium hydrogenphosphate. Biomaterials.

[31] Sun J.-S., Dong G.-C., Lin C.-Y., Sheu S.-Y., Lin F.-H., Chen L.-T., Chang W.H.-S., Wang Y.-J. (2003). The effect of Gu-Sui-Bu (Drynaria fortunei J. Sm) immobilized modified calcium hydrogenphosphate on bone cell activities. Biomaterials.

[32] Liu A., Hong Z., Zhuang X., Chen X., Cui Y., Liu Y., Jing X. (2008). Surface modification of bioactive glass nanoparticles and the mechanical and biological properties of poly(l-lactide) composites. Acta Biomater..

[33] Liu Q., de Wijn J.R., van Blitterswijk C.A. (1998). Composite biomaterials with chemical bonding between hydroxyapatite filler particles and PEG/PBT copolymer matrix. J. Biomed. Mater. Res..

[34] Khan R.A., Khan M.A., Sultana S., Nuruzzaman K.M., Shubhra Q.T.H., Noor F.G. (2010). Mechanical, degradation, and interfacial properties of synthetic degradable fiber reinforced polypropylene composites. J. Reinf. Plast. Compos..

[35] Khan R.A., Parsons A.J., Jones I.A., Walker G.S., Rudd C.D. (2011). Effectiveness of 3-aminopropyl-triethoxy-silane as a coupling agent for phosphate glass fiber-reinforced poly(caprolactone)-based composites for fracture fixation devices. J. Thermoplast. Compos. Mater..

[36] Jiang G., Evans M.E., Jones I.A., Rudd C.D., Scotchford C.A., Walker G.S. (2005). Preparation of poly(ε-caprolactone)/continuous bioglass fibre composite using monomer transfer moulding for bone implant. Biomaterials.

[37] Hasan M.S. (2012). Investigation of Coupling Agents Mediated Interfacial Integrity Improvements for Phosphate Glass Fibre Reinforced Composite for Bone Repair Applications Research. PhD Diesseration.

[38] Siparsky G.L., Voorhees K.J., Miao F. (1998). Hydrolysis of polylactic acid (PLA) and polycaprolactone (PCL) in aqueous acetonitrile solutions: autocatalysis. J. Polym. Environ..

[39] Sánchez-Vaquero V. (2010). Characterization and cytocompatibility of hybrid aminosilane-agarose hydrogel scaffolds. Biointerphases.

[40] Dong G-C., Lin F-H., Sun J-S., Yao C-H., Jiang G., Huang C-W. (2001). Biodegradability and cytocompatibility evaluation of surface modified calcium hydrogenphosphate. J. Med. Biol. Eng..

[41] Lian J.B., Stein G.S. (1992). Concepts of osteoblast growth and differentiation: basis for modulation of bone cell development and tissue formation. Crit. Rev. Oral Biol. Med..

[42] Kim H.-W., Lee H.-H., Chun G.-S. (2008). Bioactivity and osteoblast responses of novel biomedical nanocomposites of bioactive glass nanofiber filled poly(lactic acid). J. Biomed. Mater. Research A.

[43] Navarro M., Engel E., Planell J.A., Amaral I., Barbosa M., Ginebra M.P. (2008). Surface characterization and cell response of a PLA/CaP glass biodegradable composite material. J. Biomed. Mater. Res. A.

[44] Ignatius A.A., Claes L.E. (1996). *In vitro* biocompatibility of bioresorbable polymers: Poly(L,DL-lactide) and poly(L-lactide-co-glycolide). Biomaterials.

[45] Spenlehauer G., Vert M., Benoit J.P., Boddaert A. (1989). *In vitro* and *in vivo* degradation of poly(D,L lactide/glycolide) type microspheres made by solvent evaporation method. Biomaterials.

[46] Daniels A.U., Chang M.K.O., Andriano K.P., Heller J. (1990). Mechanical properties of biodegradable polymers and composites proposed for internal fixation of bone. J. Appl. Biomater..

[47] (1992). GraphPad Software, version 3.02.

